# ASO Author Reflections: Three Phases of Patient-Reported Symptom Recovery After Upper GI Cancer Surgery

**DOI:** 10.1245/s10434-026-19404-8

**Published:** 2026-03-03

**Authors:** Koichi Tomita, Naruhiko Ikoma

**Affiliations:** Department of Surgical Oncology, The University of Texas MD Anderson Cancer Center, Houston, TX USA

Patients preparing for upper gastrointestinal (UGI) cancer surgery commonly ask, “What will my recovery be like?” Yet practical, data-driven benchmarks based on longitudinal patient-reported outcomes (PROs) have been limited. Traditional indicators such as length of stay or complication rates do not capture the symptom and functional burdens that shape patients’ lived experience, making it difficult to set expectations and plan supportive care.

In a prospective cohort of 143 patients undergoing esophagectomy, gastrectomy, or pancreatectomy, we used the newly validated MD Anderson Symptom Inventory for UGI Surgery (MDASI-UGI-Surg) instrument^1,2^ to quantify symptom burden from baseline through postoperative month (POM) 6.^3^ Most symptoms peaked on postoperative day (POD) 3; pain, fatigue, sleep disturbance, drowsiness, and dry mouth were the most severe symptoms, and interference with general activity, work, and enjoyment of life was greatest. Symptom profiles were generally similar across three organ groups. Recovery followed a consistent three-phase pattern: rapid improvement from POD3 to POD14, a plateau from POD14 to POM1, and continued improvement through POM6, with fatigue persisting longer than other symptoms. We defined symptom recovery as a mean score ≤ 3 for both the top five symptoms and the top three interference items. Cumulative recovery was 64.8% at POM1, 78.9% at POM3, and 90.8% at POM6.

These PRO trajectories can help clinicians counsel patients more precisely about expected postoperative symptoms and recovery after UGI cancer surgery. In addition, routine, brief PRO monitoring embedded in perioperative workflows could enable earlier recognition of delayed recovery during the plateau phase and prompt targeted supportive care. Next, we will conduct deeper analyses—such as differences by procedure, surgical approach, and complications—to refine risk stratification and symptom-specific guidance. We will also develop and validate a short-form MDASI-UGI-Surg instrument for electronic health record implementation, expand to multicenter and multilingual settings, and test PRO-guided interventions to improve recovery and patient-centered outcomes.
